# Health Status of the Elderly and Its Influence on Their Activities of Daily Living in Shangrao, Jiangxi Province

**DOI:** 10.3390/ijerph16101771

**Published:** 2019-05-19

**Authors:** Rudan Xu, Xueqing Zhou, Shiling Cao, Boshu Huang, Chiyu Wu, Xiaojun Zhou, Yuanan Lu

**Affiliations:** 1 School of Public Health Sciences and Jiangxi Province Key Labooratory of Preventive Medicine, Nanchang University, Nanchang 330000, Jiangxi, China; xrd215162@163.com (R.X.); cc08162019@163.com (S.C.); huangboshu92@163.com (B.H.); yunhuanzijue@126.com (C.W.); 2 School of Foreign Language, South China University of Technology, Guangzhou 510000, Guangdong, China; zxq392215223@gmail.com; 3 Department of Public Health Sciences, University of Hawaii at Mānoa, 1960 East-West Road, Honolulu, HI 96822, USA

**Keywords:** elderly, long-term care (LTC), health status, activities of daily living (ADL)

## Abstract

To investigate the activities of daily living (ADL) and influencing factors, this survey study was conducted in Shangrao of Jiangxi. A total of 1087 elderly subjects in a long-term care (LTC) program participated in this study and their physical function, cognitive ability, self-rated health, and chronic disease were recorded during March 2017 and April 2018. The ADL scale was used to evaluate the health status of the elderly. F-test and multiple linear regression showed that the average ADL of the participants was 15.12 ± 17.59. The incidence of visual and verbal impairment was 68.6% and 14.1%, respectively. Over 74% of the elderly had severe cognitive impairment; and the prevalence of chronic disease was 84.5%. Multivariate analysis revealed that age, education, BMI (Body Mass Index), low income, verbal and cognitive ability, visual status, health self-evaluation, and some chronic diseases were related to self-care ability (*p* < 0.05). In summary, this study revealed that the ADL score is lower in this region and identified several influencing factors. These new findings will be useful for the local government to enhance the current LTC program for the elderly population.

## 1. Introduction

Jiangxi covers 23 districts, 66 counties, and 11 county-level cities including Shangrao city. These cities share similar economic and social conditions. At present, China is launching a long-term care insurance system, which requires individual provinces to select one of its cities as a pilot area to test for this program. Shangrao was selected as the pilot area in Jiangxi for the care insurance in 2017. Our research, which mainly assesses the activities of daily living (ADL) of the elderly who participated in long-term care insurance, is a part of this pilot program.

According to the Sixth Population Census in 2010, the number of elderly people aged 60 years and older in China increased to 178 million, reaching 13.26% of the total population [1]. Based on the data released by the National Bureau of Statistics of China, in 2016, the number of elderly people aged 60 years and above increased to nearly 231 million, accounting for 16.7% of the total population. The elderly population aged 65 years and above has now reached 150 million, accounting for 10.8% of the total population [2]. The Jiangxi Statistical Yearbook shows that the number of elderly people over the age of 60 years in the province reached 6,095,700 by the end of 2014, accounting for 13.45% of the total population [3]. China has become an aging society. Along with this demographic development, the number and proportion of the elderly who receive long-term care due to chronic degenerative diseases is increasing. This has become a serious public health crisis in China today.

To confront its aging society, China introduced a long-term care insurance system in 2016 [4]. Shangrao, Jiangxi province is one of the first pilot areas for practicing this national long-term care insurance system. ADL is the core indicator used to evaluate the long-term care needs of the elderly [5]. This indicator has been approved by WHO (World Health Organization) and is recommended for geriatric epidemiological studies on the elderly [6]. Understanding the activities of daily living of the elderly is of great significance to improve the health conditions and self-care ability of this demographic, as well as to lessen the burden placed on family caregivers. Thus, this study has the potential to impact public policy, as these findings can be useful to the government to formulate pension-related policies [7]. This study analyzes the physical function, cognitive ability, self-rated health, chronic disease status, and other dimensions of elderly people who receive long-term care, and explores the impact of these factors on the elderly ADL, which is important for the further development of the long-term care insurance.

## 2. Materials and Methods

Jiangxi province is located in southeast inland China. The main topography of Jiangxi province consists of hilly and mountainous regions as well as valley and plains. The soil composition is both red and yellow and the land is not fertile. Agricultural production is mainly based on rice farming. Jiangxi is one of the main grain production areas in China. Since it is an agricultural-based province with a low economic foundation, Jiangxi is a relatively underdeveloped region.

### 2.1. Study Population

From March 2017 to March 2018, we surveyed a total of 1087 elderly persons (aged 60 years and above) who received long-term care services in Xinzhou district (23), Guangfeng district (79), Shangrao county (38), Yushan county (48), Hengfeng county (61), Yiyang county (64), Qingshan county (73), Dexing city (114), Wuyuan county (69), Wannian county (75), Yugan county (61), and Poyang county (382). The study subjects covered 12 counties (cities and districts) within the jurisdiction of Shangrao, a city of Jiangxi province. The inclusion criteria included residents who (a) had an official household registration, (b) received long-term care services, and (c) were aged 60 years or above at the time of the survey. The exclusion criteria are as follows: those who refused to participation in investigation and physical examination, and those who lived beyond the areas being investigated. A total of 1109 cases were investigated, but only 1087 participants were enrolled because 22 cases were informed but still rejected. The effective rate of investigation was 98.02%.

### 2.2. Survey Content

This study used the ADL scale, an internationally accepted measurement of long-term health status [6]. The main contents of the questionnaire cover six different measures of health. The first is the basic demographic information of a respondent including age, gender, status of minimum social security and low income, family with no child, education level, marital status, BMI [8] (BMI = weight (kg)/height (m^2^)), old-age care type, and type and number of chronic illnesses. The second measure of health is the ADL; the specific score was expressed by utilizing the Barthel Index of Activities of Daily Living [9]. This scale evaluates the respondent’s ability to perform the activities of eating, bathing, dressing (washing face, brushing teeth, brushing hair, shaving), and 10 project items, including using the toilet. In addition, this scale records their ability to move from bed to a chair, to walk 45 m on the ground, and to go upstairs and downstairs. Among these 10 projects, each can be divided into four functional levels of 0, 5, 10, and 15 points, with a total score of 100 points according to whether the subject needs assistance and by how much. The higher the score, the stronger the independence and the smaller the dependence. Participants who score more than 60 points are considered self-sufficient. Those who score 60 to 40 points need some help. Those who score 40 to 20 points need a lot of assistance and those who score 20 points or less are considered completely unable to care for themselves. The third metric is physical function. This includes visual and verbal ability. The elderly who have normal vision and verbal ability include those whose functions have slightly declined but who do not have diminished communication in daily life. “The impaired” refers to those whose function had declined and thus experienced lowered verbal ability. The fourth metric includes the health self-evaluation (self-rated health) of “good health”, “general health”, or “poor health”. The fifth measure is cognitive ability, which uses the Mini-Mental State Examination (MMSE) [10]. This scale includes the following seven aspects: time orientation, location orientation, immediate memory, attention and computational power, delayed memory, language, and visual space. The scale is measured from 0 to 30. A score of 27–30 indicates normal cognitive function. A score of 21–26 suggests mild cognitive impairment. Those who score 10–20 may have moderate cognitive impairment. A score of 0–9 may denote the presence of severe cognitive dysfunction. The last measurement is chronic disease. This includes the existence of chronic disease, as well as the type and number of the diseases encountered (co-morbidity).

### 2.3. Survey Method

The survey was completed by door-to-door and face-to-face questions with uniformly trained and qualified investigators. The survey was started in March of 2017 and completed by 27 April 2018. The investigation environment was quiet, and the investigators were responsible for explaining the connotation of each of the investigation projects, while strictly controlling the quality to ensure the accuracy of the data.

The main contents of the questionnaire include six different health indicators: the first is basic demographic information, the second is the ADL, the third is physical function, the fourth is the health self-evaluation, the fifth is cognitive ability, which uses the Mini-Mental State Examination (MMSE), and the last measurement is chronic disease. Among these measurements, ADL was measured using the internationally accepted ADL scale, and the specific score was represented by the Barthel Index of Activities of Daily Living. The simple operation intelligence state questionnaire was used for the cognitive ability.

### 2.4. Statistical Methods

Data were recorded with the Epidata 3.1 database (version 3.1, CDC, Atlanta, GA, USA) and SPSS19.0 statistical software (SPSS Inc., Chicago, IL, USA) was used for data collation and statistical analysis. The measurement data are mainly described by mean and standard deviation, while the counting data are described by relative logarithm such as rate and composition ratio. The main methods of statistical inference included F-test, while single factors analysis was used to examine the effect of individual independent variables on ADL and multiple linear regression analysis was used to study the effect of multivariate interactions on ADL. Bilateral *p* < 0.05 was considered statistically significant.

### 2.5. Ethical Statements

This study was approved by the Ethics Committee of Jiangxi CDC (Center for Disease Control and Prevention) (Project Identification Code: ZGRSJXFGS015). Informed consent information was attached to each questionnaire and introduced before the surveys.

## 3. Results

### 3.1. Demographic Characteristics

A total of 1087 elderly residents gave consent and participated in this study. The general information about these participants is summarized in Table 1. Among the 1087 subjects, 614 males accounted for 56.5% and 473 females accounted for 43.5%, with an average age of 77.75 ± 8.12 years old (range 60 to 100 years). Only 27 respondents (2.5%) received a low income and about 4.3% (47 out of 1087) of them were bereft of their only child. Over 30% (341) were considered illiterate. More than 50% (671) of the participants were married. The elderly with normal BMI accounted for 39.3% (427 out of 1087) and the vast majority (>90.0%) chose to stay at home after they retired as compared to less than 10% who choose to stay in the pension institution. The ADL score of the elderly who received long-term care was 15.12 ± 17.592. The older the age, the lower the ADL score (*p* < 0.001). Furthermore, the higher the education level, the lower the ADL score (*p* < 0.05). Gender, low insurance and low income, loss of independence, marital status, BMI, and old-age care showed no significant difference in ADL scores among the elderly population studied (*p* > 0.05) (Table 1).

### 3.2. The Effect of the Health Status of the Elderly Who Received Long-Term Care on ADL

Among the 1087 elderly subjects aged 60 years or above who participated in long-term care insurance, 31.4% (*n* = 341) of participants had normal vision and the rest (746) had a visual impairment. The majority of the responders (934 out of 1087) had normal verbal ability while only 14.1% had a verbal impairment. The ADL score of the visually impaired was 13.12 ± 15.717 and the ADL score of the verbal impaired was 4.67 ± 10.477. A statistically significant difference was observed in the ADL among the following variables: vision and verbal ability (*p* < 0.001).

The ADL score of the elderly who rated themselves as “good-health” was found to be 22.41 ± 17.881, while 75.7% (823) considered their health status to be poor. The ADL score of the elderly who had normal cognitive ability was 25.71 ± 21.633, as compared to the majority (>74.0%) who had severe cognitive impairment and 19.3% with moderate cognitive impairment. Statistically significant differences were observed in the ADL among the variables of health self-evaluation and cognitive ability (*p* < 0.01).

Of the 1087 elderly people aged 60 years or above who participated in long-term care insurance, 918 had chronic disease, showing a prevalence rate of 84.5% (Table 2). Among these subjects, 281 elderly suffered from three or more chronic diseases, also known as co-morbidity. As shown in Table 3, the ADL score of the elderly who suffered one chronic disease was 17.41 ± 17.339. The top five major chronic diseases were hypertension (42.8%), cerebral infarction (33.1%), cerebral hemorrhage (14.9%), senile dementia (12.5%), and diabetes (12.0%). Different types of chronic diseases had different effects on the ADL. The ADL scores of the elderly with hypertension, cerebral infarction, and senile dementia were significantly lower (*p* < 0.001).

### 3.3. Influencing Factors Related to ADL

Analysis was performed by using the ADL score of the elderly who received long-term care as a dependent variable while age, gender, low income, bereavement, education level, marital status, BMI, old-age modes, type and number of chronic diseases, verbal ability, self-rate health, and vision and cognitive ability were used as independent variables. Multiple linear regression analysis was performed and the methods of assigning independent variables are shown in Table 3. Using the forward method to include variables, the inclusion level was 0.05. The results showed that older age, higher BMI index, higher education level, more chronic diseases, poor self-rated health, verbal ability impairment, cognitive impairment, visual impairment, and low income states all were associated with seniors who had poor activities of daily living scores (Table 4).

## 4. Discussion

The health status of the elderly is largely influenced by the economic development of the region [11]. Shangrao city is an economically underdeveloped area and the people’s living standards are low. With the increase of age, physical condition worsens, and basic self-care ability becomes weaker [12]. This study mainly focused on the elderly who can participate in the long-term care insurance program in Shangrao city, Jiangxi province. China, representing a special population group living in economically underdeveloped areas. The participants of this study had an average age of 77.75 ± 8.12 years with a large portion being over 80 years (45.6%). Most subjects lived at a general economic level with very few living in poverty (2.5%) (those depending on subsistence allowances and low income) with very limited education. It is very interesting to note that the vast majority of the elderly preferred to stay at home rather than in the pension institution. In addition, most of the subjects had some degree of visual impairment/loss and suffered one or more types of chronic disease; 25.9% of them had three or more chronic diseases at the same time. Our study showed that five types of chronic disease are commonly detected in the elderly in this region, including hypertension, cerebral infarction, cerebral hemorrhage, Alzheimer’s disease, and diabetes. Health self-evaluation revealed that 2.5% of the participants rated their health as good, 21.8% rated it as average, and 75.7 rated it as poor.

Using ADL and MMSE scales, this study revealed that the elderly group had a poor Dalit—an average ADL of only 17.4 ± 17.3 points. By using the MMSE assessment, this study showed that nearly all the elderly (>97%) had some degree of cognitive impairment, with 74.2% exhibiting a severe level of impairment. Compared with the Dabie Mountains of Anhui Province, which has the same economic conditions, this group of elderly people was different from ordinary elderly people in that their ADL and MMSE abilities were very low [13,14]. Compared to the general elderly population in China, the participant group had a higher prevalence of chronic disease (84.5% vs. 75.1%) [15] and higher visual loss rate (68.6% vs. 15.9%) [16]. In addition, 25.9% of the elderly participants suffered three or more chronic diseases at the same time, as compared to 11.7% in the general elderly population [17]. A higher rate of main chronic diseases was also detected in the participant group as compared to the general elderly population, including the rates of hypertension (42.8% vs. 30.1%) [15], cerebral infarction (33.1% vs. 7.8%) [17], cerebral hemorrhage (14.9% vs. 2.14%) [18], Alzheimer’s disease (12.5% vs. 2.2%) [19], and diabetes (12.0% vs. 6.6%) [19].

Findings from this study indicate the need for more care to be provided to the elderly population, increasing the current support and assistance in order to improve the health status of the elderly population including the need for the modification of the current public policies. Their situation fully meets the requirements of China’s current long-term care insurance scheme, and they should be compensated with relevant insurance funds to ensure their daily needs. The new findings obtained from this study would be helpful and important not only for promoting the establishment of an improved standard for long-term care insurance for the elderly population, but also for pointing out the critical improvements needed in the long-term care insurance system in Jiangxi province. In addition, the findings from this study strongly suggest that the pilot program in Shangrao needs to be extended to the entire elderly population in Jiangxi province.

The multivariate analysis of the ADL score among the elderly also demonstrated that older age, higher BMI, more chronic illnesses, economic difficulties (low income family), higher education, poor health self-evaluation, lack of language skills, visual loss, and cognitive problems lead to lower ADL scores. These variables are therefore determined to be all factors that affect ADL. These findings are consistent with current notions from many existing literature reports: (1) with the increase of age, the daily activity ability is weakened [20,21,22,23]. It is clear that with the decline in physical function of the elderly with age, the ADL score is lowered. (2) The greater the BMI, the lower the ADL score, which is consistent with related research conducted both in China and abroad [24,25]. BMI has become an internationally recognized standard for assessing health status, as well as an important factor influencing physical health [26]. Obesity can not only lead to costly consequences for the individual and the state [27,28], but it also seriously affects the ADL (i.e., dressing, eating, and bathing) and daily life instrumental activities of daily living (IADLs), such as preparing meals, doing housework, and shopping [29,30,31]. Obesity also greatly increases the risk of losing self-care ability in daily life [32]. (3) Chronic disease is also an important factor affecting the ADL score. Our study showed that the number of chronic diseases is closely related to the ADL score; the more chronic diseases, the lower the ADL score, which is consistent with the existing literature [33,34]. In this study, 84.5% of the elderly had one or more chronic illness conditions. Several research groups have recently reported that a variety of chronic diseases coexist for a long time, which could have a greater impact on the daily living ability of the elderly [35,36,37]. It has been noted that chronic disease is the main risk factor for disability among the elderly [38], as physical disability will naturally influence ADL. Another risk factor associated with chronic diseases of the elderly is long-term medication, which can have a detrimental effect on the vision, awareness, and mental state of the elderly. This can seriously affect the cognitive ability of the elderly, causing their ADL to be limited or even lost [39].

Additional factors associated with ADL include financial difficulties (subsistence allowances and low income), higher education level, lack of language ability, loss of vision, physical health dysfunction, poor self-rated health, and some cognitive problems. There may also be interactions between these factors. For example, a previous report indicated that there is a relationship between aging and a higher prevalence of chronic diseases [40]. In addition, the longer the disease course, the more complications, and the more types of diseases [41,42]. Overweight and obesity are also risk factors for chronic diseases, as they are associated with the development of hypertension and diabetes [41,42].

## 5. Conclusions

In summary, when a long-term care insurance system and implementation plan is developed, there is a need to fully consider the impact of relevant factors on ADL. In addition to providing a certain amount of care insurance compensation for the elderly, the long-term care plan should also help the elderly to control their weight and reduce their BMI through a series of measures such as health education, health promotion, and behavioral intervention. Furthermore, it is important and necessary for the local government to provide financial aid to those in economic difficulties, to help them overcome difficult situations, to provide physical training programs to help reduce language disability and loss of vision, and to provide personalized health care and health management plan interventions for different chronic illnesses in order to reduce complications. Thereby, the health of the elderly can be maintained, the ability of daily life will be enhanced, and the quality of life will be improved.

## Figures and Tables

**Table 1:** Basic information and activities of daily living (ADL) scores of the elderly who received long-term care.

Item	Group	No.	%	$\mathrm{ADL}\;(\bar{x}\;\pm \;s)$	95% CI		t/F	*p*
					Lower Limits	Upper Limits		
Gender	Male	614	56.5	14.76 ± 17.78	13.35	16.17	0.76	0.45
	Female	473	43.5	15.58 ± 17.36	14.01	17.15		
Age	60–64	66	6.1	20.38 ± 15.02	16.69	24.07	5.85	<0.001
	65–69	124	11.4	15.77 ± 18.14	12.54	18.99		
	70–74	196	18.0	17.81 ± 21.53	14.77	20.84		
	75–79	205	18.9	16.71 ± 18.44	14.17	19.25		
	≥80	496	45.6	12.54 ± 15.18	11.2	13.88		
Low income	Yes	27	2.5	9.63 ± 9.09	6.04	13.22	3.07	<0.001
	No	1060	97.5	15.26 ± 17.74	14.19	16.33		
Bereaved parents	No	1040	95.7	15.05 ± 17.50	13.99	16.12	0.59	0.56
	Yes	47	4.3	16.60 ± 19.70	10.81	22.38		
Education	Illiterate	341	31.4	17.51 ± 17.07	15.69	19.33	3.79	0.01
	PS	281	25.9	15.96 ± 19.31	13.69	18.23		
	MS	221	20.3	13.05 ± 17.74	10.7	15.41		
	HS	156	14.4	12.18 ± 14.45	9.89	14.46		
	≥College	88	8.1	13.58 ± 17.42	9.89	17.27		
Married	Yes	631	58.0	14.41 ± 16.86	13.09	15.72	1.58	0.12
	No	456	42.0	16.11 ± 18.54	14.40	17.81		
BMI	≤18.5	538	49.5	14.61 ± 16.99	13.17	16.05	0.67	0.57
	18.5~24	427	39.3	15.95 ± 18.83	14.16	17.74		
	24~28	89	8.2	15.11 ± 16.15	11.71	18.51		
	>28	33	3.0	12.73 ± 14.26	7.67	17.78		
Total		1087	100.0	15.12 ± 17.59	14.07	16.17		

CI: confidence interval, PS = primary school, MS = middle school, HS = high school, BMI = body mass index.

**Table 2:** The effect of the health status of the elderly who received long-term care on ADL.

	Project	*n*	%	ADL	F	*p*
Self-rated health	Good	27	2.5	22.41 ± 17.881	18.500	<0.001
	General	237	21.8	20.55 ± 21.719		
	Bad	823	75.7	13.32 ± 15.805		
Cognitive ability	Normal	28	2.6	25.71 ± 21.63	47.53	<0.001
	Mild CI	42	3.9	31.07 ± 27.29		
	Moderate CI	210	19.3	23.76 ± 20.25		
	Severe CI	807	74.2	11.67 ± 14.44		
Chronic disease	No	169	15.5	23.08 ± 23.82	23.97	<0.001
	Yes (1)	357	32.8	17.41 ± 17.34		
	Yes (2)	280	25.8	11.46 ± 14.28		
	Yes (≥3)	281	25.9	11.07 ± 14.14		
Main type	Hypertension	465	42.8	12.96 ± 15.27	12.41	<0.001
	Cerebral infarction	360	33.1	12.69 ± 15.32	10.32	<0.001
	Cerebral hemorrhage	162	14.9	12.01 ± 12.32	5.99	0.02
	Senile dementia	136	12.5	10.22 ± 16.15	12.18	<0.001
	Diabetes	130	12.0	12.62 ± 15.72	3.00	0.08

CI = cognitive impairment.

**Table 3:** Multiple linear regression analysis of independent variable assignment.

Independent Variables	Independent Variable Assignment Methods
Gender	Female = 0; Male = 1
Age	“60~ years old” = 0; 65~ = 1; 70~ = 2; 75~ = 3; 80~ = 4
Low-income group or not	Not = 0; Low-income group = 1
Bereaved parents or not	Not = 0; Yes = 1
Education level	Illiterates = 1; Primary school = 2; Middle school = 3; High school = 4; University or above = 5
Whether have spouses	Not = 0; Yes = 1
BMI	18.5 or lower = 0; 18.5~ = 1; 24~ = 2; 28 = 3
Old-age modes	Pension institution = 0; Stay at home = 1
Number of chronic diseases	No = 0; One kind = 1; Two kinds = 2; Three kinds or above = 3
Verbal ability	Impaired = 0; Normal = 1
Health self-evaluation	Good = 0; General = 1; Bad = 2
Vision	Impaired = 0; Normal = 1
Cognitive ability	Normal = 0; Mild impairment = 1; Moderate impairment = 2; Severe impairment = 3

**Table 4:** Multiple linear regression analysis of factors influencing ADL.

Independent Variables	Non-Standardized Coefficient		Standardized Regression Coefficient	t	*p*
	B	Standard Error			
Age (years)	0.24	0.05	0.81	5.06	<0.001
BMI (kg/m^2^)	0.57	0.11	0.49	5.35	<0.001
Health self-evaluation	−3.23	0.98	−0.25	−3.31	<0.001
No. chronic diseases	−3.59	0.51	−0.30	−7.03	<0.001
Verbal ability	−4.71	1.47	−0.24	−3.21	<0.001
Vision: normal or not	4.93	1.09	0.12	4.53	<0.001
Low income	19.45	2.92	0.83	6.67	<0.001
Cognitive ability	−4.57	0.78	−0.73	−5.88	<0.001
Education level	−0.81	0.40	−0.10	−2.02	0.04
